# Characterization and Dissolution Study of Chitosan Freeze-Dried Systems for Drug Controlled Release

**DOI:** 10.3390/molecules14114370

**Published:** 2009-10-30

**Authors:** Roberto Ruiz-Caro, María Dolores Veiga-Ochoa

**Affiliations:** 1 Departamento de Farmacia y Tecnología Farmacéutica, Facultad de Farmacia, Universidad Complutense de Madrid, Plaza Ramón y Cajal s/n, 28040-Madrid, Spain; 2 Unidad de Biotransformaciones Industriales, Parque Científico de Madrid, Tres Cantos, Madrid, Spain

**Keywords:** caffeine, chitosan, freeze-dried systems, swelling and dissolution behaviour, controlled release

## Abstract

Freeze-dried systems (L) comprising chitosan (CS) and caffeine (CAF) have been developed for oral administration. Different proportions of CS and CAF have been used in the preparation of the systems. Hot stage microscopy (HSM), differential scanning calorimetry (DSC) and X-ray diffraction powder have been used to characterize the systems prepared. X-ray diffraction patterns showed that there were no interactions between CAF and CS molecules within the freeze-dried systems and the crystallinity of CAF was decreased. Swelling and dissolution tests were carried out in two different media (demineralized water and pH progressive medium) in order to establish their influence over CAF/CS system behaviour. Characteristic swelling behaviour of freeze-dried CS systems (imbibition and dissolution processes) was influenced by the proportions of CS and CAF in the formulations, and by the nature of the medium due to the pH-dependent solubility of CS. Release of CAF from lyophilized systems was conditioned by the swelling process and it should be possible to obtain a CAF/CS binary system with a specific time for total drug release including concrete proportions of both components. Furthermore, the freeze-drying process allowed us to obtain feasible systems for controlled release of CAF until the total amount of drug was released.

## 1. Introduction

Time-controlled oral drug delivery systems offer several advantages over immediate-release dosage forms, including the minimization of fluctuations in drug concentrations in the plasma and at the site of action over prolonged periods of time, resulting in optimized therapeutic effectiveness and reduced side effects, a reduction of the total dose administered (while providing similar therapeutic effects) and a reduction of the administration frequency, leading to improved patient compliance [1]. The choice of excipients in order to obtain controlled release drug delivery systems is of paramount importance. Adequate excipients and methodologies allow one to obtain the desired drug delivery rate.

Chitosan (CS) is known as an excellent material for drug preparation. Chemically, it is a linear polycationic copolymer of β(1–4) linked 2-acetamide-2-deoxy-β-D-glucopyranose and 2-amino-2-deoxy-β-D-glucopyranose obtained from deacetylation of chitin, the second most abundant natural polysaccharide [2]. Chitin is an important constituent of the exoskeletons of animals, especially crustaceans (crabs, shrimps and lobster), molluscs and insects. It is also the principal fibrillar polymer in the cell wall of certain fungi [3,4]. CS has been gaining increasing importance in the pharmaceutical field owing to its good biocompatibility, low toxicity and biodegradability [5,6]. This polymer has found wide applicability in conventional pharmaceutical devices as a potential formulation excipient, some of which include binding, disintegrating and tablet coating properties [7]. CS is a weak base with a pK_a_ value of the D-glucosamine residue of about 6.2–7.0 and, therefore, it is insoluble at neutral and alkaline pH values. Although the solubility of CS in inorganic acids is limited when compared with its solubility in common organics acids, it forms salts with inorganic and organic acids such as hydrochloric acid, acetic acid, glutamic acid, and lactic acid. In acid medium, the amine groups of the polymer are protonated, resulting in a soluble, positively charged polysaccharide (RNH_3_^+^) that has a high charge density. Moreover, CS can form gels by interacting with different types of divalent and polyvalent anions [8,9,10,11,12,13]. CS shows swelling ability in contact with aqueous media [14] and mucoadhesivity in the gastrointestinal tract [15] and in the oral cavity [16,17,18]. In consequence, CS can be used in the preparation of systems which are suitable for achieving sustained release of either hydrophilic or lipophilic drugs [19]. Lee *et al*. studied tablets with CS/Carbopol^®^971NF interpolymer complexes and theophylline and they observed that the release mechanism of theophylline from a matrix tablet consisting of the interpolymer complex varied depending on the pH of the medium used in the preparation of the complex. Thereby, when the pH of the medium used in the preparation of the complex was 4, a high controlled release of theophylline was obtained at pH 6.8 for 12 hours. However, when the release medium was at pH 1.2 no influence of the pH of the medium used in the preparation of the complex is showed [20]. As CS can form gels by interacting with different types of divalent anions [12], CS diacetate was cross-linked with Zn^2+^ ions to yield ionotropically crosslinked polymeric matrices, thus, tablets loaded with CAF showed a sustained release of drug in 500 minutes and there was observed effectively in a zero-order manner [21]. 

Moreover, this polysaccharide has been investigated for its possible application as dissolution enhancer of poorly water-soluble drugs [22,23,24]. Also, CS crosslinked with glutaraldehyde or tripolyphosphate has proven to be suitable for colonic drug delivery [25]. Oosegi *et al.* have developed Eudragit L100-coated CS-succinyl-prednisolone microparticles as a prodrug of prednisolone for controlled release to achieve a targeted oral delivery system against inflammatory bowel disease. Thus there was suppressed release at stomach pH, but it exhibited gradual release at intestinal pH values [26]. CS was also investigated as a coating polymer for obtaining sustained release of several water-soluble drugs. Thereby, coated microtubular halloysite (aluminosilicate mineral with a hollow tubular structure) with CS cross-linked with glutaraldehyde for the sustained release of diltiazem hydrochloride and propranolol hydrochloride was investigated by Levis and Deasy. They demonstrated that a high amount of only CS and an increase in the coating thickness yield a high sustained release of the diltiazem hydrochloride to pH 6.8 for 8 hours. Moreover, the results for the dissolution profile obtained at pH 6.8 indicated that the cross-linking treatment with 10% of glutaraldehyde produced some additional delayed release, as was also observed at pH 3.2 [27]. Lin *et al.* synthesized particles with a crosslinked poly(CS-N-isopropylacrylamide/methacrylic acid–methyl methacrylate) thermal-sensitive and core-shell type copolymer for its potential use in drug targeting. With an increase of the weight ratio of methacrylic acid/methyl methacrylate or a decrease of the shell thickness of particles, the swelling ratio of the sample increased and the amount of CAF loaded into the particles was in proportion to the equilibrium swelling ratio. Thereby, the drug that was really loaded into the particles (20%–30%) was trapped and protected from release [28]. Huang *et al*. formulated CS microspheres crosslinked with chlorpheniramine maleate (CPM)-resinates embedded in glutaraldehyde in order to obtain a controlled release of the water-soluble drug. Thereby, they demonstrated controlled release of CPM in simulated gastric fluid (SGF) and simulated intestinal fluid (SIF) without enzymes being about 60% by 1 h in SGF, and about 100% by 3 h in SIF. The retardation effect increased when the crosslinking extent and CS to resin ratio increased [29].

In this study, CAF was chosen as model water-soluble drug. CAF (1,3,7-trimethyl-3,7-dihydro-1*H*-purine-2,6-dione) [30] is a psychostimulant drug and it has a very limited use indicated in fatigue and primary newborn apnea [31]. Due to its water solubility it was used as a model drug to evaluate its release from formulations with different excipients able to modulate the release of it or other hydrosoluble drugs [32,33,34,35].

With these backgrounds, the aim of this research is to develop an easy and simple method for making CS-based systems able to control release of water-soluble drugs, such as CAF, in the gastrointestinal tract. 

## 2. Results and Discussion

Different CAF/CS systems, using acetate buffer for dissolving CAF and CS have been prepared by a freeze-drying process. Their compositions are showed in Table 1. In order to determine the interaction degree obtained between CS and CAF, the lyophilized systems and the raw materials employed for their preparation have been characterized in solid state by the following techniques: X-ray diffraction powder, hot stage microscopy and differential scanning calorimetry. Swelling and dissolution behaviour of all CAF/CS lyophilized systems have been studied in order to know the influence of CS swelling/dissolution behaviour on the CAF controlled release from the systems. Also, swelling behaviour of blank systems (B), prepared without CAF, has been evaluated.

**Table 1:** Proportions of caffeine and chitosan in solutions used in the preparation of lyophilized systems.

Formulations	Composition (g/100mL)	
	CAF	CS
L1	1	1
L2	1	2
L3	1	3
L4	1	4
L5	1	5
L6	2	1
L7	2	2
L8	2	3
L9	2	4
L10	2	5
B1	–	1
B2	–	2
B3	–	3
B4	–	4
B5	–	5

### 2.1. X-Ray Diffraction Analysis

**Figure 1:**
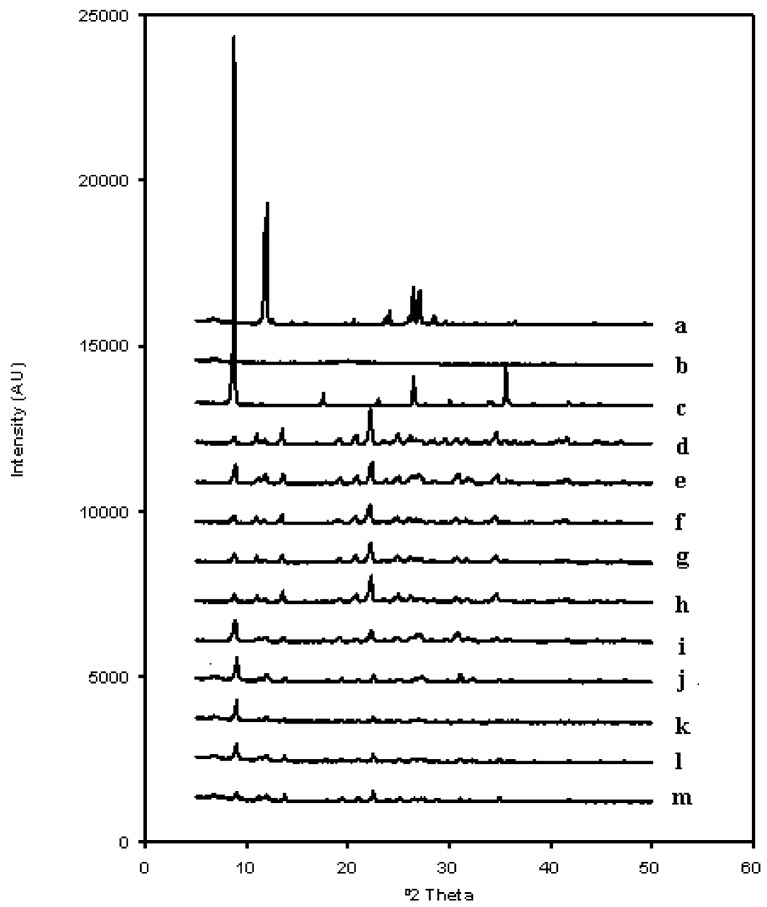
X-ray diffraction patterns of caffeine (a), chitosan (b), sodium acetate (c) and freeze-dried formulations L1 (d), L2 (e), L3 (f), L4 (g), L5 (h), L6 (i), L7 (j), L8 (k), L9 (l), L10 (m) prepared with acetate buffer pH = 4.5.

Figure 1 shows the diffraction patterns of pure CAF, CS, sodium acetate (SA) and all formulations prepared. The diffraction pattern of CAF showed its characteristic peaks at 11.9º, 26.5º and 27.1º 2θ. CS did not exhibit any diffraction peak, due to its amorphous nature. SA (used for the preparation of all lyophilized systems) displayed a diffraction pattern with its characteristic peaks at 8.8º, 17.6º, 26.5º and 35.6º 2θ. All types of lyophilized systems were crystalline and they showed similar diffraction patterns, where CAF peaks were displayed with less intensity. Also, the SA peak at 8.8º 2θ appeared in all the freeze-dried systems. Moreover, new different peaks appeared at 13.6º and 22.3º 2θ, which can be due to the formation of a new crystalline structure in the freeze-drying process, because the new peaks cannot be attributed to any solid raw material used in the preparation of the samples. 

### 2.2. Thermal Analysis

Thermomicroscopic observation of pure CAF at room temperature revealed that the sample was constituted of small irregular crystalline particles. In the range of 127–140 ºC, the sample experienced a partial sublimation and recrystallization, giving way to longer needle and laminar shaped crystals. In the range of 230–234 ºC the melting of the drug took place. The melted product exhibited at higher temperatures evaporation processes (320–350 ºC). 

The sample of SA was constituted by crystalline, dark and irregular shaped particles. During heating, darkening of the particles was detected in the 60–90 ºC range. Then, the sample remained unchanged until its melting range (323–326 ºC) was reached. No decomposition ocurred, because when the melted sample was cooled, the formation of spectacular crystals was observed at the thermo-microscope.

Under the microscope, CS was found to be constituted of irregular shaped particles. During heating, a slight darkning was observed in the range between 40–140 ºC. Then, the sample remained unchanged until the temperature of 270 ºC was reached. At that point, a decomposition process, without previous fusion, ocurred. 

All the CAF/CS freeze-dried systems (L1 → L10) showed similar aspect and thermomicroscopic behaviour. All the samples exhibited crystalline and irregular particles of different sizes. During heating, in the 60–160 ºC range a modification in the shape of the particles, due to loss of solvents used in the preparation of the systems, was shown. At higher temperatures (120–160 ºC) the characteristic sublimation and recrystallization processes of CAF were observed. A slight darkness of the sample, due to decomposition of CS, was noticed at 260 ºC. When the temperature reached the range of 320–326 ºC, the melting of SA contained in the sample was produced. No interaction between SA melted and dark particles of carbonized CS was detected. 

**Figure 2:**
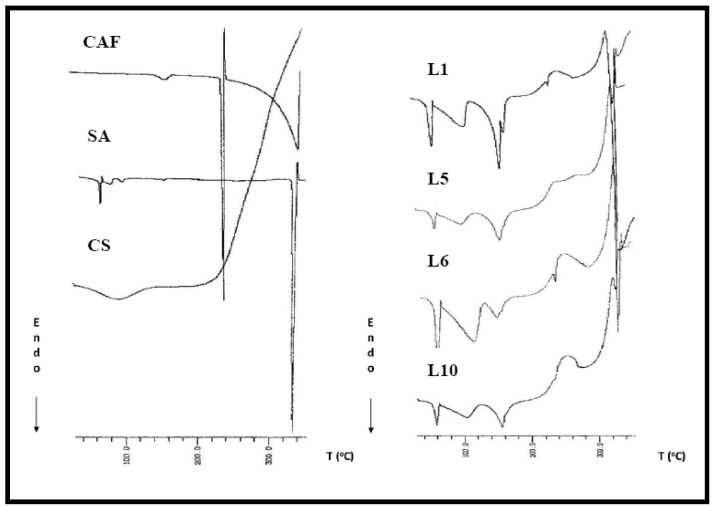
DSC curves of caffeine (CAF), chitosan (CS), sodium acetate (SA)and lyophilized formulations L1, L5, L6 and L10.

The DSC curve of pure CAF (Figure 2) showed three endothermic peaks, that can be explained by the hot stage microscope observations. The first one (157.7 ºC) corresponds to the characteristic sublimation-recrystallization process of CAF (new crystal like needles were observed on the HSM). The second peak (233.5 ºC) was the melting of the sample, and the last one (340 ºC) corresponding to the evaporation of melted CAF (also detected by the HSM). 

SA displayed a DSC curve (Figure 2) with some little peaks in the 40–100 ºC range corresponding to the loss of recrystallization solvent (darkening of the particles was detected by HSM). Then, recovery of the baseline ocurred. When the 320–340 ºC range was reached, a sharp endothermic peak corresponding to the melting (325.4 ºC) was apparent. 

The CS thermogram shows a typical polysaccharide behaviour [36,37], with two distinct degradation stages. The first one is a wide peak which starts at 30 ºC and continues up to 150 ºC corresponding to a dehydration process. The second stage started at 220 ºC and an elevation in the baseline, corresponding to combustion of the sample, was observed (it was checked by HSM observation). No endothermic melting peak was displayed due to the amorphous state of the CS. The DSC curves of all lyophilized systems were similar, and for this reason, only the DSC curves of four systems (L1, L5, L6 and L10) have been shown in Figure 2.

The different thermal events reflected in these curves have been explained by HSM. The first and second endothermic peak (corresponding to the ranges from 40 to 65 ºC and 65 to 120 ºC) were associated with the loss of the solvent used in the preparation of samples (acetic acid) and the CS dehydration process. These events were detected by thermomicroscopic observation as a total modification in the shape of the sample particles. The third endothermic peak (120–180 ºC) corresponded to sublimation-recrystallization of CAF. At temperatures above 200 ºC an elevation in the baseline, due to combustion of CS, was displayed in all the freeze-dried systems, and only in DSC curves of L1 and L6 (systems with low proportion of CS) a little endothermic peak corresponding to the melting of CAF was shown. The last endothermic peak reflected the SA melting process. 

### 2.3. Swelling Test

**Figure 3:**
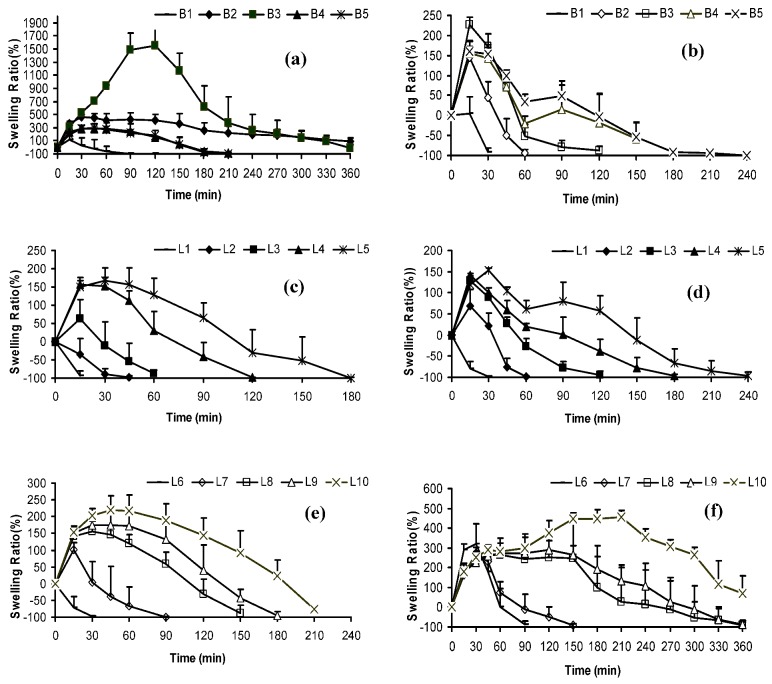
Swelling Ratio of chitosan and freeze-dried systems in demineralized water (a, c and e) and in progressive pH medium (1.5 → 4 → 6.8) (b, d and f).

Weight evolution of all freeze-dried systems was assayed in demineralized water and in progressive pH medium and represented as swelling ratios (Figure 3). 

Each positive swelling ratio value indicated that, at this time, the swollen system weight was higher than the dry system weight (t = 0). On the contrary, each negative swelling ratio value indicates that the weight of swollen system was lower than the weight of the dry system (t = 0). When t = 0, the swelling ratio value is 0 in all systems, due to the application of the equation shown in the Experimental section below. 

In general, two steps were detected in the swelling behaviour of all systems. The first step corresponded to an increase in the weight of the systems because CS imbibed aqueous media, and the second one showed a weight loss due to CS dissolution. Swelling behaviour was conditioned by: CS and CAF proportions and medium nature. Swelling ratio data at specific times (30, 60, 120 and 180 minutes) in demineralized water and in progressive pH medium are reported in Table 2. The specific time intervals were chosen in order to observe the different swelling ratio data due to the pH medium changes. 

**Table 2:** Swelling Ratios of all lyophilized systems at specific time intervals (30, 60, 120 and 180 minutes) in demineralized water and in progressive pH medium (1.5 → 4 → 6.8).

Formulations	Swelling Ratio (% ± SD)								
	Demineralized water				Progressive pH medium				
	30 min	60 min	120 min	180 min		30 min	60 min	120 min	180 min
B1	22 ± 127	−56 ± 77	−96 ± 7	–		−92 ± 11	–	–	–
B2	466 ± 37	416 ± 94	407 ± 100	258 ± 119		44 ± 40	−95 ± 9	–	–
B3	524 ± 55	932 ± 19	1557 ± 316	621 ± 316		172 ± 33	−53 ± 30	−88 ± 10	–
B4	286 ± 52	284 ± 86	159 ± 99	−57 ± 33		143 ± 33	−20 ± 18	−19 ± 73	–
B5	286 ± 15	271 ± 47	181 ± 74	−78 ± 18		153 ± 18	35 ± 19	−4 ± 57	−91 ± 8
L1	–	–	–	–		−99 ± 2	–	–	–
L2	−89± 14	–	–	–		21 ± 31	−99 ± 2	–	–
L3	−11 ± 65	−88 ± 10	–	–		89 ± 3	−27 ± 20	−94 ± 10	–
L4	153 ± 23	31 ± 50	−98 ± 1	–		101 ± 12	20 ± 8	−38 ± 28	−97 ± 5
L5	168 ± 34	128 ± 45	−31 ± 64	−100 ± 1		153 ± 6	61 ± 20	57 ± 37	−65 ± 33
L6	−98 ± 3	–	–	–		318 ± 102	2 ± 95	–	–
L7	4 ± 62	−66 ± 56	–	–		274 ± 8	73 ± 56	−48 ± 50	–
L8	157 ± 29	121 ± 25	−33 ± 45	–		236 ± 33	264 ± 62	253 ± 185	100 ± 210
L9	174 ± 33	172 ± 32	40 ± 75	−94 ± 10		227 ± 22	275 ± 76	288 ± 50	192 ± 64
L10	204 ± 21	217 ± 47	144 ± 51	24 ± 46		254 ± 49	283 ± 21	373 ± 8	448 ± 45

The swelling behaviour of blank systems (B1 → B5) indicated that the higher swelling and dissolution was obtained from B3, which has a middle concentration of CS (3%). In blank systems with lower amounts of CS (B1 and B2) the water penetrated easily and the CS was dissolved with barely swelling (Figure 3a). In the case of systems with high concentrations of CS (B4 and B5), water was absorbed in the outer layers of the system forming a compact gel and the systems slowly dissolved from outside to inside. Under progressive pH conditions the behaviour is different (Figure 3b) due to the fact CS is soluble in acid medium [2] and thus the swelling ratio values in all the systems are lower to the corresponding ones obtained in water, because a part of the CS was dissolved and it cannot absorb medium (Table 2).

This change in the swelling behaviour due to a change of medium, affected all the blank systems and the turning point in terms of the swelling still corresponds to system B3. Furthermore, the blank systems which remained undissolved at 60 minutes (B4 and B5), when pH medium changed from 1.5 to 4, showed an increase in their weights, because the deprotonation produced in CS amino groups caused a decrease in its solubility and, in consequence, the swelling process was increased.

Figure 3(c and e) depict the swelling ratios of CAF lyophilized systems in demineralized water. In general, the presence of the drug impeded the characteristic swelling behaviour of CS, because a competitive process between CS and CAF molecules towards water molecules was produced, so CAF was dissolved in water and the CS swelling process was attenuated. 

The increase in the concentration of CAF from 1% (Figure 3c) to 2% (Figure 3e) (L1 → L6, L2 → L7, L3 → L8, L4 → L9 and L5 → L10) produced an increase in their swelling ratio values as reported in Table 2. This fact could be attributed to a slower diffusion rate of CAF solution from the systems to the medium, because of the higher CAF concentration in these systems (L6, L7, L8, L9 and L10).

Swelling ratios of CAF lyophilized systems in progressive pH medium are displayed in Figure 3(d and f). Swelling ratio values of systems with low proportions of CAF (L1 → L5) (Figure 3d) were slightly lower, or equal, to the corresponding blank systems, because CAF did not disturb the characteristic swelling/dissolution behaviour of CS in progressive pH medium. On the contrary, the systems with a higher proportion of CAF (L6 → L10) showed a different swelling behaviour (Figure 3f), because these systems again showed the competitive process between CAF and CS towards medium molecules. CAF was dissolved, and its concentrated dissolution could not dissolve CS. But this concentrated dissolution remained in the systems allowing the swelling of CS. 

For this reason, all systems with 2% of CAF captured higher amounts of medium than systems with 1% of CAF, or the blank systems. Furthermore, in the case of the systems with 2% of CAF, the remains of the sample were lasted for more prolonged time and a new change of pH medium was produced. 

**Figure 4:**
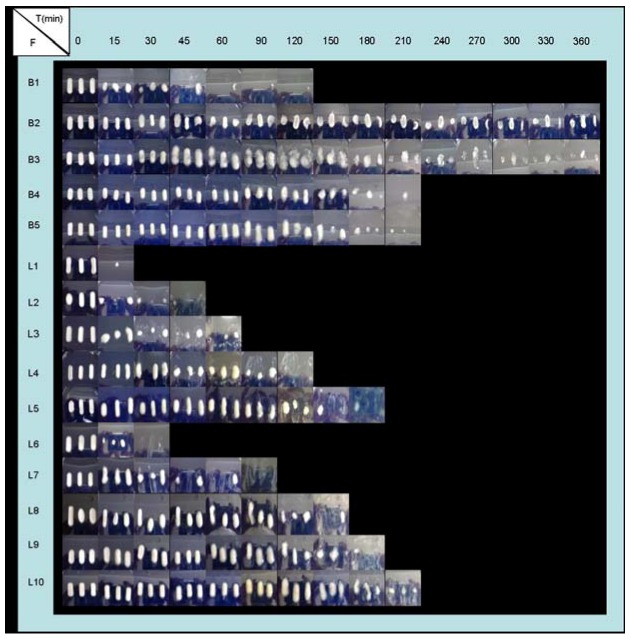
Appearance evolution of freeze-dried formulations (F) during swelling tests in demineralized water.

**Figure 5:**
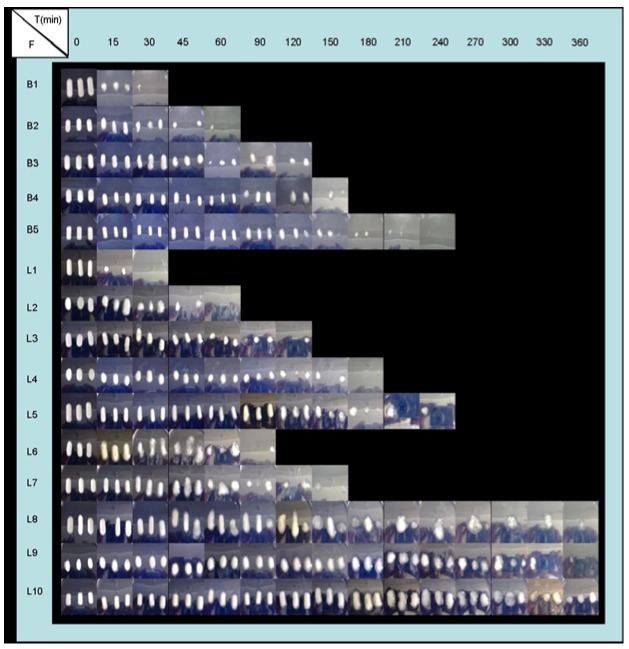
Appearance evolution of freeze-dried formulations (F) during swelling tests in progressive pH medium (1.5 → 4 → 6.8).

So, when the pH medium increased from 1.5 to 4 (at 60 minutes) the deprotonation of CS occurred and again, the swelling process prevailed over the CS dissolution process. The explanation of swelling behaviour of all systems studied is in agreement with the evolution of their appearance as shown by the systems over the time course of the experiments (Figure 4 and Figure 5). 

### 2.4. Dissolution Study

**Figure 6:**
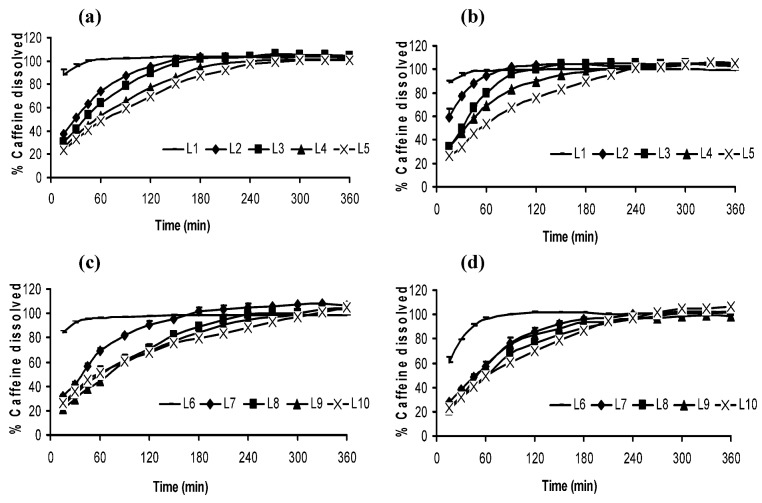
Dissolution profiles of CAF freeze-dried systems in demineralized water (a and c) and in progressive pH medium (1.5 → 4 → 6.8) (b and d).

Dissolution profiles of CAF from lyophilized systems are displayed in Figure 6. The graphs reveal that drug dissolution process was mainly conditioned by the proportion of CS. The systems with low amounts of CS (L1 and L6) showed faster drug release profiles because of their low consistency. On the contrary, systems with high CS proportions were more compact and therefore, they maintained their shape for more prolonged time. Thus, these systems showed drug controlled release (L2 – L5, L7 – L10) where 100% of CAF was dissolved in 1.5 to 6 hours in demineralized water and progressive pH medium. The amount of CAF in the systems conditioned slightly the drug release. The presence of CAF modulated the CS swelling behaviour, as explained above, and therefore, the CAF dissolution was also modulated. Thereby, the systems with 2% of CAF delayed the imbibition of the medium and in consequence, drug release rate was also decreased.

**Table 3:** Caffeine released in both media from lyophilized systems at measured time intervals of 60 and 120 minutes.

Formulations	CAF release (% ± SD)			
	demineralized water		progressive pH medium	
	60 minutes	120 minutes	60 minutes	120 minutes
L1	100.96 ± 0.62	102.79 ± 0.28	98.80 ± 0.34	99.85 ± 0.33
L2	73.64 ± 2.13	94.89 ± 1.84	94.68 ± 1.07	103.37 ± 1.36
L3	63.53 ± 1.06	89.30± 0.78	79.94 ± 0.91	100.00 ± 0.37
L4	52.64 ± 0.53	77.00 ± 1.02	69.25 ± 0.90	89.90 ± 1.02
L5	48.19 ± 0.76	69.56 ± 0.56	53.53 ± 2.68	75.56 ± 2.81
L6	96.52 ± 0.59	97.89 ± 0.65	96.31 ± 1.30	101.61 ± 0.64
L7	68.93 ± 2.82	90.26 ± 3.41	56.99 ± 4.13	85.80 ± 3.39
L8	49.71 ± 1.71	70.69 ± 1.23	48.30 ± 4.91	75.97 ± 5.09
L9	44.76 ± 0.59	68.32 ± 0.68	57.70 ± 1.22	83.18 ± 0.98
L10	51.03 ± 5.17	67.36 ± 7.70	49.50 ± 2.45	70.30 ± 2.64

In all the cases, 1% CAF systems released drug faster than 2% CAF systems, due to their different porosity. The samples analyzed show a different pore size distribution, i.e. the CS sample show a monomodal distribution around 13 μm, which accounts for the 60% of the porosity, being the total porosity around 70%. On the other hand, the L10 sample is characterized by a bimodal pore size distribution at 13 μm and 4 μm; in this case only a 10% of porosity can be attributed to the bigger pores while pores below 5 mm add up to 38% to complete a total porosity value (48%) much lower than that described for CS samples (70%), so in the system L1, low amounts of CAF and CS (70% porosity and 13 μm of size pore), the total release of CAF, in both dissolution media, was reached in 1 hour. On the contrary, in the system with higher proportions of CS and CAF, L10, (48% porosity with pores around 13 μm and 4 μm) the diffusion process of CAF molecules through the system was more difficult. 

The influence of dissolution medium in CAF release is obtained by comparing the dissolution profiles of every lyophilized system in both media. No clear differences were noticed in systems with a low proportion of CS (L1 and L6), because the fast CS dissolution, explained in the swelling study, allowed the practically instantaneous drug dissolution. The systems with intermediate amounts of CS and low proportion of CAF (L2, L3 and L4) displayed dissolution curves influenced by the nature of the medium, because of swelling/dissolution CS pH-dependent [1,2]. Data of drug release at 60 minutes (Table 3) revealed faster drug dissolution in progressive pH medium (acid pH at this time) with regard to demineralized water. The influence of dissolution medium composition in CAF release is low in the systems with high proportions of CS and CAF, because the systems were very compact and dissolution was modulated by swelling process. The systems with high proportions of CS and CAF (L5, L8, L9 and L10) showed dissolution profiles very similar in both media, but the data of drug released at 60 and 120 minutes revealed that these systems were slightly most soluble in acid medium. From dissolution data it is possible to deduce that L5, L8, L9 and L10 are the most suitable systems to control CAF release.

## 3. Experimental

### 3.1. Materials

Caffeine (Lot: 014K0026) was purchased from Sigma (St. Louis, MO, USA). Chitosan with a deacetylation degree of 97.0%and a viscosity of 92.0 cSt (Lot: 8826900003) was supplied by Nessler (Madrid, Spain). All other reagents were analytical grade. 

### 3.2. Preparation of Caffeine/Chitosan Freeze-Dried Systems (L)

CAF, previously sieved, with a particle size < 100 µm, was dissolved in sodium acetate buffer (pH 4.5) [38]. Then, CS (particle size < 100 µm) was added to the CAF solution. The system was carefully mixed on a magnetic stirrer until a homogeneous solution was obtained. In order to prepare lyophilized systems, different solutions of CAF/CS were dosified into PVC casts and then they were freeze-dried (Lio-Labor^®^; Telstar, Barcelona, Spain) for 48 hours reaching a freezing temperature, a sublimation temperature and a sublimation pressure into a chamber of −45 ºC, from −45 to 25 ºC and 4.54 × 10^−4^ atm, respectively. Blank lyophilized systems (non loaded with CAF) (B1 → B5) were also prepared to compare them to systems with CAF in the swelling study. 

### 3.3. Characterization of Caffeine/Chitosan Freeze-Dried Systems (L)

#### 3.3.1. X-ray diffraction analysis

The powder X-ray diffraction patterns of pure materials and all CAF/CS lyophilized systems were recorded by using an automated Philips X’Pert X-ray diffractometer. Samples were irradiated with monochromatized Cu-Kα radiation and analyzed between 2*θ* angles of 5° and 40°. The voltage, the current, and the time per step were 40 mV, 55 mA and 1 s, respectively. A software package attached with the diffractometer was used to calculate the peak heights of all diffraction patterns (CAI, DRX, UCM). Powder X-ray diffraction patterns were measured in order to evaluate the crystalline/amorphous character of pure ingredients untreated and CAF/CS lyophilized systems. 

#### 3.3.2. Differential scanning calorimetry

DSC curves of the pure materials and all freeze-dried systems were recorded on a Mettler TA 3000 differential scanning calorimeter (model DSC 20). About 5–10 mg of sample were placed in a pinholed aluminium sample pan with lid and heated in atmospheric air at a rate of 10 °C/min between 30 and 350 °C. The instrument was periodically calibrated with a standard sample of indium.

#### 3.3.3. Hot stage microscopy

About 1 mg of sample was placed on a microscopic slide with cover and heated at a rate of 2 °C/min on a Kofler stage and samples were studied between 30 and 350 °C. Microscopic examinations were carried out by using a Thermogalen microscope fitted with the Kofler stage.

#### 3.3.4. Swelling test

Swelling of all systems prepared was evaluated as weight gain when suspended then in an aqueous medium at 37 ± 0.1 ºC. This test was carried out in a preliminary study in demineralized water and then, due to these systems have been designed for oral administration and they should cover the gastrointestinal tract, the swelling and the dissolution tests were also carried out in progressive pH medium (acid medium pH 1.5 the first hour, followed by the addition of NaOH 10M until pH reached a value of 4.0 until 3 hours. A new addition of NaOH 10M modified pH reaching a value of 6.8 until the 6 hours). The maximum duration of assay was 6h. At specific time intervals, the samples were removed from test medium and were blotted with filter paper to absorb excess liquid on sample surface. At the same time, photographs were taken with a digital camera (Fujifilm^®^ Finepix A345 4.1 Megapixels) to observe the behaviour and changes of all systems in contact with the media. The swelling ratio (SR%) of every sample was calculated following the expression [39]:

SR% = [(Ls − Ld)/Ld] × 100% (1)

where Ls and Ld were the weights of the swollen and dried samples, respectively.

#### 3.3.5. Dissolution study

A Sotax AT-7 dissolution apparatus with paddles was employed to carry out all of the tests. The volume of the dissolution medium, experimental temperature, and paddle speed were 1,000 mL of either demineralized water or progressive pH medium, 37 ± 0.1 °C, and 50 rpm, respectively. 100 mg of CAF (previously sieved, size < 100 μm), or its equivalent amount from lyophilized systems, were used for all dissolution studies. The maximum duration of assay was 6 h. Samples were withdrawn at measured time intervals and filtered with a Whatman^®^ filter paper (type 42). The quantity of dissolved CAF was determined at a wavelength of 271.5 nm (pH 1.5) and at 272.0 nm (pH 4, pH 6.8 and demineralized water), using in all cases a Beckman DU-7 spectrophotometer and three replicates of each dissolution assay were carried out. It was tested previously that there was no change in the λ_max_ of CAF due to the presence of CS.

#### 3.3.6. Porosity

A Hg intrusion porosimetry study was carried out using a Micromeritics AutoPore III 9410 porosimeter. The samples where analyzed between 0.10 and 60.000 psia, using a filling pressure of 1 psia with equilibration times of 10 seconds.

## 4. Conclusions

The combination of a suitable carrier (CS) and a simple methodology (freeze-drying process) has allowed us to obtain solid formulations for drug controlled release. Swelling and dissolution data allow us to conclude that release of CAF from lyophilized systems was only conditioned by the swelling processes, since the lack of interaction between CAF and CS of freeze-dried systems as proven by X-ray diffraction and thermal analysis did not condition the drug controlled release. In consequence, it should be possible to obtain a CAF/CS binary system which displays a controlled swelling with a specific time for total drug release including concrete proportions of CS. Although CS showed swelling/dissolution pH-dependent, CAF controlled release were maintained, because a modification in pH medium could influence only in a slight way the swelling behaviour and drug dissolution from our lyophilized systems.

## References

[1] Streubel A., Siepmann J., Bodmeier R. (2006). Gastroretentive drug delivery systems. Expert Opin. Drug Deliv..

[2] Illum L. (1998). Chitosan and its use as a pharmaceutical excipient. Pharm. Res..

[3] Tomihata K., Ikada Y. (1997). *In vitro* and *in vivo* degradation of films of chitin and its deacetylated derivatives. Biomaterials.

[4] Felt O., Buri P., Gurny R. (1998). Chitosan: A unique polysaccharide for drug delivery. Drug Dev. Ind. Pharm..

[5] Agnihotri S.A., Mallikarjuna N.N., Aminabhavi T.M. (2004). Recent advances on chitosan-based micro- and nanoparticles in drug delivery. J. Control. Rel..

[6] Sinha V.R., Singla A.K., Wadhawan S., Kaushik R., Kumria R., Bansal K., Dhawan S. (2004). Chitosan microspheres as a potential carrier for drugs. Int. J. Pharm..

[7] Singla A.K., Chawla M. (2001). Chitosan: some pharmaceutical and biological aspects—an update. J. Pharm. Pharmacol..

[8] Amiji M.M., Patel V.R. (1996). Preparation and characterization of freeze-dried chitosan-poly(ethylene oxide) hydrogels for site-specific antibiotic delivery in the stomach. Pharm. Res..

[9] Knapczyk J., Krowczynski L., Krzek J., Brzeski M., Nurnberg E., Schenk D., Struszczyk H., Skjak-Braek G., Anthonsen T., Sandford P. (1989). Requirements of chitosan for pharmaceutical and biomedical applications. Chitin and Chitosan–Sources, Chemistry, Biochemistry, Physical Properties and Applications.

[10] Sandford P.A., Skjak-Braek G., Anthonsen T., Sandford P. (1989). Chitosan: commercial uses and potential applications. Chitin and Chitosan–Sources, Chemistry, Biochemistry, Physical Properties and Applications.

[11] Errington N., Harding S.E., Varum K.M., Illum L. (1993). Hydrodynamic characterization of chitosans varying in degree of acetylation. Int. J. Biol. Macromol..

[12] Fukuda H. (1980). Polyelectrolyte complexes of chitosan with sodium carboxymethylcellulose. Bull. Chem. Soc. Jpn..

[13] Fukuda H., Kikuchi Y. (1978). Polyelectrolyte complexes of chitosan with sodium carboxymethyldextran. Bull. Chem. Soc. Jpn..

[14] Ubaidulla U., Khar R.K., Ahmad F.J., Sultana Y., Panda A.K. (2007). Development and characterization of chitosan succinate microspheres for the improved oral bioavailability of insulin. J. Pharm. Sci..

[15] Hassan E.E., Gallo J.M. (1990). A simple rheological method for the *in vitro* assessment of mucin-polymer bioadhesive bond strength. Pharm. Res..

[16] Lueßen H.L., Lehr C.M., Rentel C.O., Noach A.B.J., de Boer A.G., Verhoef J.C., Junginger H.E. (1994). Bioadhesive polymers for the peroral delivery of peptide drugs. J. Control. Rel..

[17] Lueßen H.L., de Leeuw B.J., Langemeÿer M.W., de Boer A.G., Verhoef J.C., Junginger H.E. (1996). Mucoadhesive polymers in peroral peptide drug delivery. VI. Carbomer and chitosan improve the intestinal absorption of the peptide drug buserelin *in vivo*. Pharm. Res..

[18] Luesßen H.L., Rentel C.O., Kotzé A.F., Lehr C.M., de Boer A.G., Verhoef J.C., Junginger H.E. (1997). Mucoadhesive polymers in peroral peptide drug delivery. IV. Polycarbophil and chitosan are potent enhancers of peptide transport across intestinal mucosae *in vitro*. J. Control. Rel..

[19] Remuñán-López C., Portero A., Vila-Jato J.L., Alonso M.J. (1998). Design and evaluation of chitosan/ethylcellulose mucoadhesive bilayered devices for buccal drug delivery. J. Control. Rel..

[20] Lee M.H., Chun M.K., Choi H.K. (2008). Preparation of Carbopol/chitosan interpolymer complex as a controlled release tablet matrix: Effect of complex formation medium on drug release characteristics. Arch. Pharm. Res..

[21] Aiedeh K.M., Taha M.O., Al-Hiari Y., Bustanji Y., Alkhatib H.S. (2007). Effect of ionic crosslinking on the drug release properties of chitosan diacetate matrices. J. Pharm. Sci..

[22] Zerrouk N., Mennini N., Maestrelli F., Chemtob C., Mura P. (2004). Comparison of the effect of chitosan and polyvinylpyrrolidone on dissolution properties and analgesic effect of naproxen. Eur. J. Pharm. Biopharm..

[23] Park K.M., Bae J.W., Joung Y.K., Shin J.W., Park K.D. (2008). Nanoaggregate of thermosensitive chitosan-Pluronic for sustained release of hydrophobic drug. Colloids Surf. B. Biointerfaces.

[24] Lim Soo P., Cho J., Grant J., Ho E., Piquette-Miller M., Allen C. (2008). Drug release mechanism of paclitaxel from a chitosan-lipid implant system: Effect of swelling, degradation and morphology. Eur. J. Pharm. Biopharm..

[25] McConnell E.L., Murdan S., Basit A.W. (2008). An Investigation into the Digestion of Chitosan (Noncrosslinked and Crosslinked) by Human Colonic Bacteria. J. Pharm. Sci..

[26] Oosegi T., Onishi H., Machida Y. (2008). Novel preparation of enteric-coated chitosan-prednisolone conjugate microspheres and in vitro evaluation of their potential as a colonic delivery system. Eur. J. Pharm. Biopharm..

[27] Levis S.R., Deasy P.B. (2003). Use of coated microtubular halloysite for the sustained release of diltiazem hydrochloride and propranolol hydrochloride. Int. J. Pharm..

[28] Lin C.L., Chiu W.Y., Lee C.F. (2005). Preparation of thermoresponsive core-shell copolymer latex withpotential use in drug targeting. J. Colloid Interface Sci..

[29] Huang R.G., Schwartz J.B., Ofner C.M. (1999). Microencapsulation of chlorpheniramine maleate-resin particles with crosslinked chitosan for sustained release. Pharm. Dev. Technol..

[30] Moffat A.C. (1986). Clarke´s Isolation and Identification of Drugs in Pharmaceuticals, Body Fluids, and Post-mortem Materials.

[31] Villa L.F. (2004). Medimecum.

[32] Wu N., Wang L.S., Tan D.C., Moochhala S.M., Yang Y.Y. (2005). Mathematical modelling and *in vitro* study of controlled drug release via a highly swellable and dissoluble polymer matrix: polyethylene oxide with high molecular weights. J. Control. Rel..

[33] Ainaoui A., Siepmann J., Bodmeier R., Vergnaud J.M. (2001). Calculation of the dimensions of dosage forms with release controlled by diffusion for *in vivo* use. Eur. J. Pharm. Biopharm..

[34] Hu Z., Mawatari S., Shimokawa T., Kimura G., Yoshikawa Y., Shibata N., Takada K. (2000). Colon delivery efficiencies of intestinal pressure-controlled colon delivery capsules prepared by a coating machine in human subjects. J. Pharm. Pharmacol..

[35] Lagarde D., Batejat D., Sicard B., Trocherie S., Chassard D., Enslen M., Chauffard F. (2000). Slow-release caffeine: a new response to the effects of a limited sleep deprivation. Sleep.

[36] De Lima M.S., Freire M.S., Fonseca J.L., Pereira M.R. (2009). Chitosan membranes modified by contact with poly(acrylic acid). Carbohydr. Res..

[37] Synytsya A., Synytsya A., Blafková P., Ederová J., Spevacek J., Slepicka P., Král V., Volka K. (2009). pH-controlled self-assembling of meso-tetrakis(4-sulfonatophenyl)porphyrin-chitosan complexes. Biomacromolecules.

[38] (2005). Real Farmacopea Española (RFE).

[39] Haupt S., Zioni T., Gati I., Kleinstern J., Rubinstein A. (2006). Luminal delivery and dosing considerations of local celecoxib administration to colorectal cancer. Eur. J. Pharm. Sci..

